# Artificial Intelligence and Its Effect on Dermatologists’ Accuracy in Dermoscopic Melanoma Image Classification: Web-Based Survey Study

**DOI:** 10.2196/18091

**Published:** 2020-09-11

**Authors:** Roman C Maron, Jochen S Utikal, Achim Hekler, Axel Hauschild, Elke Sattler, Wiebke Sondermann, Sebastian Haferkamp, Bastian Schilling, Markus V Heppt, Philipp Jansen, Markus Reinholz, Cindy Franklin, Laurenz Schmitt, Daniela Hartmann, Eva Krieghoff-Henning, Max Schmitt, Michael Weichenthal, Christof von Kalle, Stefan Fröhling, Titus J Brinker

**Affiliations:** 1 Digital Biomarkers for Oncology Group (DBO) National Center for Tumor Diseases (NCT) German Cancer Research Center (DKFZ) Heidelberg Germany; 2 Skin Cancer Unit German Cancer Research Center (DKFZ) Heidelberg Germany; 3 Department of Dermatology, Venereology and Allergology University Medical Center Mannheim University of Heidelberg Heidelberg Germany; 4 Department of Dermatology University Hospital Kiel University of Kiel Kiel Germany; 5 Department of Dermatology University Hospital Munich (LMU) Munich Germany; 6 Department of Dermatology University Hospital Essen University of Duisburg-Essen Essen Germany; 7 Department of Dermatology University Hospital Regensburg Regensburg Germany; 8 Department of Dermatology University Hospital Würzburg University of Würzburg Würzburg Germany; 9 Department of Dermatology University Hospital Erlangen University of Erlangen Erlangen Germany; 10 Department of Dermatology University Hospital Cologne Cologne Germany; 11 Department of Dermatology University Hospital Aachen Aachen Germany; 12 Department of Clinical-Translational Sciences Charité University Medicine and Berlin Institute of Health (BIH) Berlin Germany; 13 National Center for Tumor Diseases German Cancer Research Center Heidelberg Germany

**Keywords:** artificial intelligence, machine learning, deep learning, neural network, dermatology, diagnosis, nevi, melanoma, skin neoplasm

## Abstract

**Background:**

Early detection of melanoma can be lifesaving but this remains a challenge. Recent diagnostic studies have revealed the superiority of artificial intelligence (AI) in classifying dermoscopic images of melanoma and nevi, concluding that these algorithms should assist a dermatologist’s diagnoses.

**Objective:**

The aim of this study was to investigate whether AI support improves the accuracy and overall diagnostic performance of dermatologists in the dichotomous image–based discrimination between melanoma and nevus.

**Methods:**

Twelve board-certified dermatologists were presented disjoint sets of 100 unique dermoscopic images of melanomas and nevi (total of 1200 unique images), and they had to classify the images based on personal experience alone (part I) and with the support of a trained convolutional neural network (CNN, part II). Additionally, dermatologists were asked to rate their confidence in their final decision for each image.

**Results:**

While the mean specificity of the dermatologists based on personal experience alone remained almost unchanged (70.6% vs 72.4%; *P*=.54) with AI support, the mean sensitivity and mean accuracy increased significantly (59.4% vs 74.6%; *P*=.003 and 65.0% vs 73.6%; *P*=.002, respectively) with AI support. Out of the 10% (10/94; 95% CI 8.4%-11.8%) of cases where dermatologists were correct and AI was incorrect, dermatologists on average changed to the incorrect answer for 39% (4/10; 95% CI 23.2%-55.6%) of cases. When dermatologists were incorrect and AI was correct (25/94, 27%; 95% CI 24.0%-30.1%), dermatologists changed their answers to the correct answer for 46% (11/25; 95% CI 33.1%-58.4%) of cases. Additionally, the dermatologists’ average confidence in their decisions increased when the CNN confirmed their decision and decreased when the CNN disagreed, even when the dermatologists were correct. Reported values are based on the mean of all participants. Whenever absolute values are shown, the denominator and numerator are approximations as every dermatologist ended up rating a varying number of images due to a quality control step.

**Conclusions:**

The findings of our study show that AI support can improve the overall accuracy of the dermatologists in the dichotomous image–based discrimination between melanoma and nevus. This supports the argument for AI-based tools to aid clinicians in skin lesion classification and provides a rationale for studies of such classifiers in real-life settings, wherein clinicians can integrate additional information such as patient age and medical history into their decisions.

## Introduction

Melanoma detection and classification is a challenging task but diagnostic accuracy can be enhanced using aids such as dermoscopy, which has improved the examination of pigmented and nonpigmented skin lesions with the naked eye [1-3]. The combination of dermoscopy with reflectance confocal microscopy could further increase the diagnostic accuracy of melanocytic lesions, thereby highlighting the potential of the complementary approaches [4]. A comparatively new strategy falling within the realm of computer-aided diagnosis (CAD) is the use of trained convolutional neural networks (CNNs) to analyze macroscopic images of suspicious lesions. Studies have shown that, within certain limitations and considering a purely image-based setting, artificial intelligence (AI) can achieve on par or superior performance to dermatologists [5-9], thereby highlighting its potential as a decision-support system with immediate clinical implications.

Previous studies have shown that dermatologists perform better as a group [10] or as an ensemble of human and machine [11]. An imputation analysis of the results from the International Skin Imaging Collaboration (ISIC) 2017 challenge showed an increase in the dermatologists’ performance when low-confidence decisions were replaced by a classifier’s diagnosis [12]. However, in these studies, participants’ answers were independent of each other and combined or modified retrospectively. The performance of the dermatologists with live information from CAD systems has to be investigated in more detail.

The CAD system MelaFind, intended for multispectral digital analysis of melanocytic lesions, has been evaluated in experimental settings and has shown to improve dermatologists’ sensitivity, albeit at the cost of lower specificity [13,14], which has raised concerns about its benefits [15]. AI-based systems tend to be more balanced in terms of sensitivity and specificity scores and have shown to improve the classification accuracy of nondermatologists by using a content-based image retrieval algorithm [16]. Outside of dermatology, similar studies have shown the benefit of AI [17] while also highlighting the challenges AI assistance faces, as it can be both helpful and misleading at the same time [18]. This study builds on and investigates whether live AI support in the form of a classic CNN architecture is capable of improving the accuracy and the overall diagnostic performance of experts (dermatologists) in the dichotomous image–based discrimination between melanoma and nevus.

## Methods

### Study Design

This study was conducted from January 10, 2019 (design of study) to September 27, 2019 and was inspired by the study design of Sinz et al [2]. Questionnaires were sent out on June 12, 2019 and were completed by August 12, 2019. Ethical approval was waived by the ethics committee of the University of Heidelberg, Mannheim Faculty of Medicine as all the images were open source and anonymous and all participating dermatologists automatically became part of the study group.

The set of images used for the evaluation consisted of 1200 unique dermoscopic images split into 12 nonoverlapping individual test sets containing 100 images each (50 melanomas and 50 nevi). Each individual set was randomly assigned to exactly one dermatologist who diagnosed his/her set twice—first without AI support (part I) and afterwards with AI support (part II). Images for each part were presented to the participants in 2 separate surveys containing 50 images each (again 50:50 split) to investigate a possible learning effect. Thus, every participant completed 4 surveys (2 surveys for part I and 2 surveys for part II) where images seen in part I were identical to those seen in part II. Both surveys from part I were carried out before any survey from part II. The participants received detailed instructions by email in which the survey structure and the procedure were discussed and the lesion distribution was disclosed. For part II, participants were made aware of the classifier’s performance, which was established beforehand on a separate validation set.

### Classifier Training

Images were obtained from the ISIC archive [19], with a large fraction of images coming from the HAM10000 dataset [20]. The archive is publicly accessible and contains anonymous dermoscopic images from multiple sources and various camera systems. From the available pigmented lesions, only images showing a biopsy-verified melanoma (n=1633) or nevus (n=3311) were selected (total n=4944).

As each of the 12 individual test sets consisted of 2 subsets (with 50 images each), there were 24 test subsets. A designated training set was constructed for each test subset by removing its images from the total image data, resulting in 24 training sets, each containing 4894 images (ie, 4944 – 50). The class distribution across each training set was unevenly distributed with 1608 melanoma images in contrast to 3286 nevus images. To counter the class imbalance, the set of melanoma images was duplicated. Online data augmentation was used to increase the diversity of the training data and to modify the duplicated images. Before balancing, an evenly balanced validation set was removed from each training set for calibration and validation purposes later. A detailed description of the classifier training is given in Multimedia Appendix 1.

### Classifier Integration

For AI support to be used in an effective manner, a confidence measure was displayed in addition to the binary decision (melanoma or nevus), which indicated the classifier’s confidence in its decision. The measure was obtained by mapping the classifier’s raw output probability for the predicted class into a percentage range from 0% to 100%.

Participants were made aware of the classifier’s overall performance by establishing the overall performance of all 24 classifiers on their validation set and by taking the average, which resulted in mean sensitivity, mean specificity, and mean accuracy of 78.0%, 81.0%, and 80.0% respectively.

### Electronic Survey and Participants

Fifteen participants were invited by personal invitation via their university email accounts by the principal investigator (TJB) based on previous cooperation. Three had to be excluded because one reported to not have time to do the second survey properly, and 2 did not attempt or finish the survey in the proposed timeframe, leaving 12 dermatologists (all board-certified and coauthors of this publication) from 9 German university hospitals for analysis. The Google Forms closed web-based survey was not anonymous and each participant had to disclose his or her full name and additional metadata. For part I, the dermatologists were shown images of biopsy-verified skin lesions and asked 3 mandatory questions. First, a personal rating of the image quality had to be given (excellent, good, sufficient, poor, other image problems, no image visible). Second, the participant was asked for a diagnosis of the displayed lesion with the options being benign or malignant. Third, the participant was requested to quantify his or her confidence in the decision on a scale of 0 (=very uncertain) to 10 (=very certain). Part II showed identical images and questions and was additionally labeled with the dermatologist’s previous response and the diagnosis of the CNN together with its confidence level. For details on the participants, see Multimedia Appendix 1.

### Performance and Statistical Analysis

The primary endpoint of this study was whether dermatologists’ accuracy (overall proportion of the correct predictions among the total number of examined cases) would increase with AI support with the secondary endpoint investigating how sensitivity and specificity would change (tested for significance using Wilcoxon test). Additional analyses investigated whether dermatologists experienced a learning effect when working with AI support (tested for significance using Pearson’s chi-squared test) and how they would respond to this form of computer aid. Statistical significance was considered at *P*<.05, corrected to 0.016 (Bonferroni correction for the primary and secondary endpoint) to account for multiple testing. The details on how survey answers were evaluated are listed in Multimedia Appendix 1. As a pair of classifiers was responsible for scoring the test set of one participant (one classifier for survey 1, the other for survey 2), the results of both classifiers were combined for analysis reasons so that every dermatologist had a respective counterpart. Values shown in the result sections are based on the mean of all the participants. If absolute values are shown, the denominator and numerator are approximated. In theory, every dermatologist rated the exact same number of images, but due to the quality control step, not every rating was counted. Therefore, each dermatologist ended up rating a varying number of images.

## Results

### Classification Results

In the first part of the study, the dermatologists had an overall mean sensitivity of 59.4% (95% CI 53.3%-65.5%), specificity of 70.6% (95% CI 62.3%-78.9%), and accuracy of 65.0% (95% CI 62.3%-67.6%, see Table 1).

In the second part of the study, where participants could integrate the results of the CNN-based classifier in their decision-making, their overall sensitivity increased significantly to 74.6% (95% CI 69.9%-79.3%; *P*=.003). Their mean specificity also showed a positive trend (72.4%; 95% CI 66.2%-78.6%; *P*=.54), so that the overall accuracy also increased significantly to 73.6% (95% CI 70.9%-76.3%; *P*=.002).

**Figure 1:**
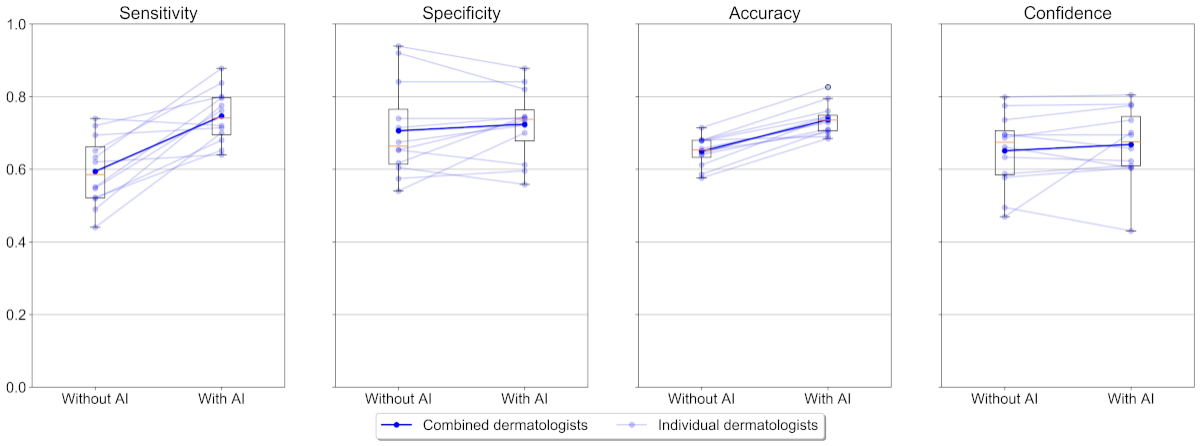
Combined and individual dermatologists’ performance without and with artificial intelligence (AI) support. Every dot represents a single participant. A line between 2 dots connects the participants' metric without AI support to the corresponding metric with AI support. Highlighted dots represent the participants combined. Boxes indicate 25th and 75th percentile while the horizontal line within shows the median (50th percentile). Whiskers indicate the data range (1.5*IQR) where points beyond are considered as outliers.

**Figure 2:**
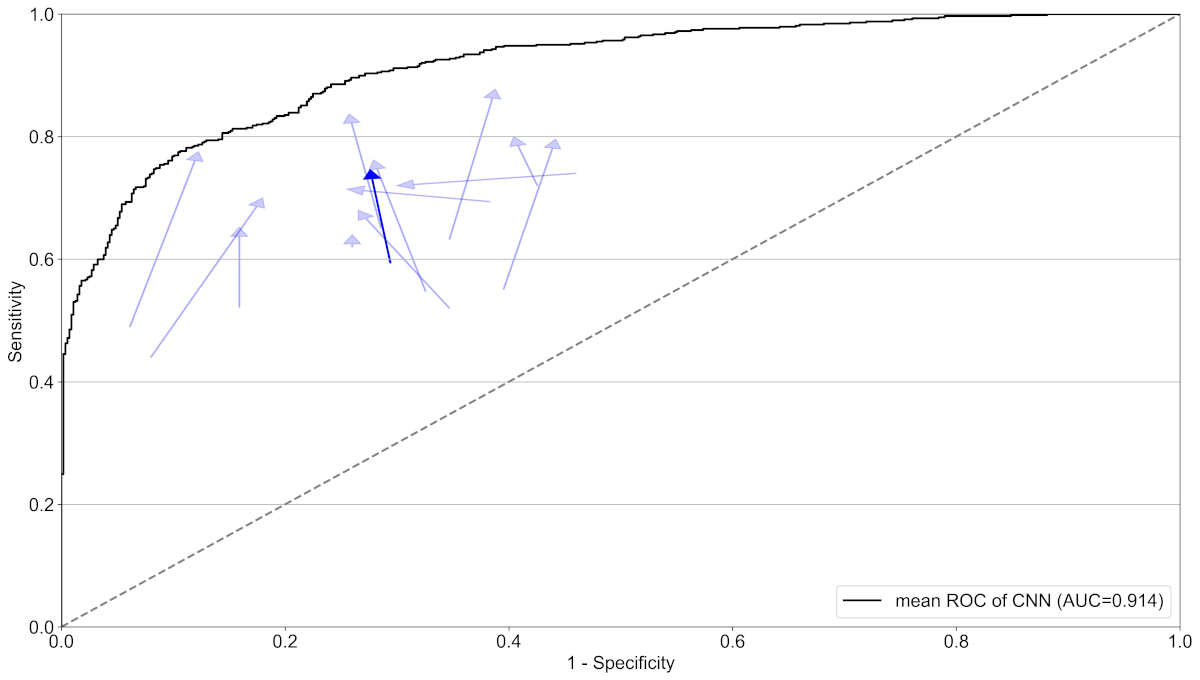
Combined and individual dermatologists’ diagnostic accuracy without and with artificial intelligence (AI) support. Diagnostic accuracy is measured using sensitivity and specificity. Arrows represent the change in the diagnostic accuracy from without AI support to with AI support. Highlighted arrows represent the participants combined. In addition, the black curve denotes the mean receiver operating characteristic curve of the classifier. ROC: receiver operating characteristic; CNN: convolutional neural network; AUC: area under the curve.

The CNN on its own had an even higher sensitivity (84.7%; 95% CI 81.9%-87.6%), specificity (79.1%; 95% CI 74.8%-83.4%), and accuracy (81.9%; 95% CI 79.7%-84.2%) on the test set, which was comparable to its performance on the validation set. The overall performance of the dermatologists and the CNN are summarized in Table 1 while individual performances are captured in Figure 1 and Figure 2, which show an overview of the various metrics for every participant with and without AI support.

**Table 1:** Overall mean performance of dermatologists without artificial intelligence (AI) support (-AI) compared to that of dermatologists with AI support (+AI) and AI on its own. Performance is split into 3 categories, that is, sensitivity, specificity, and accuracy.

Performance	AI	Dermatologist -AI	Dermatologist +AI
Sensitivity (95% CI)	84.7% (81.9%-87.6%)	59.4% (53.3%-65.5%)	74.6% (69.9%-79.3%)
Specificity (95% CI)	79.1% (74.8%-83.4%)	70.6% (62.3%-78.9%)	72.4% (66.2%-78.6%)
Accuracy (95% CI)	81.9% (79.7%-84.2%)	65.0% (62.3%-67.6%)	73.6% (70.9%-76.3%)

### Effects of the Use of the Classifier on the Decisions of the Dermatologists

The mean overall confidence of the dermatologists increased only marginally with the use of the classifier (65.0%; 95% CI 58.5%-71.6% without AI to 66.8%; 95% CI 60.2%-73.4% with AI). The confidence of the classifier in its diagnostic decisions was at 65.0% (95% CI 62.8%-67.4%). Upon detailed analysis, one can detect opposite effects on the dermatologists’ confidence and switching behavior depending on agreement or disagreement with the classifier. On average, dermatologists and classifiers agreed in 63% (59/94; 95% CI 59.4%-66.1%) of cases. Among these, 8% (7/94) of cases were diagnosed incorrectly and 55% (52/94) of cases were classified correctly (see Table 2).

**Table 2:** Distribution of the correct and incorrect predictions by classifier and dermatologists without artificial intelligence (AI) support and switching in response to AI support. Percentages displayed below show the amount of times a switch did or did not occur for dermatologists when answering part II of the survey.

Groupings		Proportion, n (%)^a^	95% CI
**Both incorrect, n=94**		7 (8)	5.5%-10.4%
	Dermatologist switched, n=7	0 (1)	0%-2.2%
	Dermatologist stayed, n=7	7 (99)	97.8%-100%
**AI correct,** **Dermatologist** **incorrect, n=94**		25 (27)	24.0%-30.1%
	Dermatologist switched, n=25	11 (46)	33.1%-58.4%
	Dermatologist stayed, n=25	14 (54)	41.6%-66.9%
**AI incorrect,** **Dermatologist** **correct, n=94**		10 (10)	8.4%-11.8%
	Dermatologist switched, n=10	4 (39)	23.2%-55.6%
	Dermatologist stayed, n=10	6 (61)	44.4%-76.9%
**Both correct, n=94**		52 (55)	52.4%-57.1%
	Dermatologist switched, n=52	0 (0)	0%-0.5%
	Dermatologist stayed, n=52	52 (100)	99.5%-100%

^a^As the mean of all the participants was taken and every participant ended up rating a varying amount of images due to the quality control step, the reported absolute values are approximations.

Of the 37% (35/94; 95% CI 33.9%-40.6%) of cases in which dermatologists and classifier came to different conclusions, 10% (10/94) of cases were diagnosed correctly by the dermatologists and 27% (25/94) of cases were diagnosed correctly by the classifier.

In cases of agreement, the mean confidence of the dermatologists increased substantially from the first part of the study to the second part of the study (from 67.0%; 95% CI 60.6%-73.5% to 79.1%; 95% CI 75.0%-83.0%, respectively). In cases of disagreement, it decreased from 61.7% (95% CI 54.6%-68.9%) in the first to 44.3% (95% CI 31.8%-56.7%) in the second part of the study. This was also reflected in the dermatologists’ switching behavior. In cases of agreement, the dermatologists basically never altered their classifications (0/59, 0%; 95% CI 0%-0.8%). In contrast, in cases of disagreement, the dermatologists altered their decision in 43% (15/35; 95% CI 30.8%-56.3%) of those cases. The dermatologists altered their diagnosis less often when they had initially diagnosed the lesion correctly (subsequently changed answer in 4/10, 39% of those cases) than when they had initially diagnosed it incorrectly (subsequently changed answer in 11/25, 46% of those cases, see Table 2). Altogether, this resulted in the observed overall increase in the correct diagnoses by the dermatologists. Out of all the occurring switches, dermatologists showed a higher willingness to switch from benign to malignant (11/15, 72%; 95% CI 53.4%-90.9%) than from malignant to benign (4/15, 28%; 95% CI 9.1%-46.6%).

The mean confidence levels of the classifier were much higher when the correct conclusion was reached (71.7%; 95% CI 69.3%-74.1%) than when the diagnosis was incorrect (34.8%; 95% CI 30.9%-38.7%). Regarding the dermatologists, a similar trend was less striking for part I (67.3%; 95% CI 61.1%-73.5% correct vs 60.8%; 95% CI 53.3%-68.2% incorrect) but became more pronounced for part II (71.8%; 95% CI 66.4%-77.1% correct vs 52.3%; 95% CI 41.0%-63.5% incorrect). A more detailed breakdown of the dermatologists’ and classifier’s confidence levels is shown in Table 3. Finally, the dermatologists altered their diagnoses in divergent cases more often when the CNN’s confidence levels were high than when they were low (mean CNN confidence levels 63.3%; 95% CI 56.4%-70.1% with subsequent switch vs 53.7%; 95% CI 46.7%-60.7% with no subsequent switch).

**Table 3:** Confidence distribution of the classifier and dermatologists for part I and part II.

Groupings		Confidence (95% CI)
**Both incorrect**		
	AI^a^	35.8% (27.6%-44.1%)
	Dermatologist part I	62.3% (54.6%-69.9%)
	Dermatologist part II	69.8% (62.4%-77.3%)
**AI correct, Dermatologist incorrect**		
	AI	65.4% (60.7%-70.2%)
	Dermatologist part I	60.8% (53.3%-68.3%)
	Dermatologist part II	46.0% (33.6%-58.5%)
**AI incorrect, Dermatologist correct**		
	AI	32.9% (28.1%-37.6%)
	Dermatologist part I	63.2% (56.3%-70.2%)
	Dermatologist part II	47.9% (36.4%-59.4%)
**Both correct**		
	AI	74.5% (72.0%-76.9%)
	Dermatologist part I	67.9% (61.5%-74.3%)
	Dermatologist part II	80.3% (76.2%-84.3%)

^a^AI: artificial intelligence.

### Learning Effect

The dermatologists received their results immediately after completing the first survey of the second part of the study. Thus, they had the opportunity to implement the lessons learned in the first AI-supported survey during the completion of the second survey in part II. A subanalysis showed that there was no detectable change in the dermatologists’ performance from the first to the second survey in part I of the study (see figure in Multimedia Appendix 2) with no significant difference between sensitivity (*P*=.50) and specificity (*P*=.76). In contrast, the dermatologists tended to perform better in the second survey of part II compared to the first survey, suggesting that they had learned how to better incorporate the CNN results into their diagnostic procedures, albeit with differences in sensitivity (*P*=.21) and specificity (*P*=.43) being still insignificant. In divergent cases, participants switched their diagnosis in 49% (8/17; 95% CI 36.2%-62.3%) of cases in survey 2, whereas they had only done so in 38% (7/18; 95% CI 21.1%-54.5%) of these cases for survey 1. The tendency to switch at the correct moments in cases of disagreement was reinforced in the second survey wherein dermatologists switched far more often when they were incorrect (5/13, 39%; 95% CI 23.0%-55.7% in the first survey vs 6/12, 52%; 95% CI 39.8%-64.3% in the second survey).

## Discussion

### Principal Results

This study provides support for the value of CNN-based deep learning algorithms as complementary diagnostic tools in skin cancer detection as each of the 12 participating dermatologists experienced increased accuracy when working together with the classifier. In particular, the sensitivity of melanoma detection was improved. The specificity of the classification did not deteriorate substantially. This is remarkable because there is usually a trade-off between sensitivity and specificity in diagnostic tests. Thus, our classifier increased the overall accuracy of melanoma classification by the dermatologists. The one-sided performance improvement could be based on the classifier’s higher confidence for the subset of melanoma images where a switch did occur coupled with the assumption that dermatologists tend to change their mind more readily toward malignant cases. Based on balanced accuracy, every physician experienced a performance boost when working with AI support. The increase in balanced accuracy varied among the participants, with participants having a lower balanced accuracy for part I, generally showing larger improvements. Interestingly, improvement was not solely determined by classifier performance as the top three most improved participants worked with a classifier performing worse than average. The observed switching rate by dermatologists when disagreeing with the classifier reinforces previous findings, which indicate physicians’ susceptibility to recommendations of decision support systems [21].

The overall confidence of the dermatologists increased substantially when there was an agreement between the classifier and the dermatologist but decreased when there was a disagreement. The increased diagnostic confidence when there was an agreement could have an impact on treatment decisions, as the confidence to excise or not to excise increases, but it is not without downsides as confidence levels also increased when both agreed but were incorrect, reinforcing the participants’ confidence in the wrong classification. The observed confidence trend when disagreeing is expected, as an opposite viewpoint can create doubts in one’s own diagnosis. This is however only beneficial when the classifier is correct but harmful when it is incorrect as it not only results in decreased confidence but also in participants switching from right to wrong. In the end, accuracy improvements were attained due to the combination of the classifier being right more often than wrong and dermatologists correctly being able to assess when to trust the classifier and when not to. Further improving such a system would therefore entail improving the classifier’s performance as well as its integration so that participants are better capable to delineate when to trust the classifier and when to trust themselves.

The dermatologists’ performance with AI support showed a nonsignificant trend of improvement between survey 1 and survey 2 for part II. This could indicate that after a single “practice session,” dermatologists had gained enough experience with the use of the classifier and adapted their diagnostic procedure in a way that yielded better performance. Further practice and more detailed analysis of the way in which dermatologists interact with the classifier may improve performance even further.

The exact reason for dermatologists to switch is difficult to pin down. As each participant was shown his or her previous answer (part I) for questions for part II, the change of mind presumably occurred because of the classifier’s answer. We cannot rule out that dermatologists would reconsider and switch on their own upon second viewing, but it is unlikely based on the fact that dermatologists almost never switched when agreeing with the classifier.

### Limitations and Further Considerations

Currently, the algorithm on its own has a higher diagnostic accuracy than the dermatologists with AI support. However, the setting in which the dermatologists had to reach their decisions in this study does not completely reflect the real life situation wherein clinicians can integrate further information into their final decisions, such as age, patient history, or lesion localization, and an unbalanced distribution of melanomas and nevi. Therefore, adding the AI classifier to the routine diagnostic procedures already in place in the clinic and exploring its effects is an interesting next step. Completely taking the physician out of the loop is questionable as neural networks are known to have robustness issues and while patients may accept the usage of CAD systems, it is tied to the condition that physicians interpret the results of such systems and are not replaced by them [22]. Furthermore, the small sample size of the participants does not reflect the experience levels encountered in the clinic (eg, no trainee doctors) but was consciously chosen to ensure conscientious participation.

The comparatively low sensitivity attained by the participants could be due to the nature of the survey question, as participants were asked for a classification (melanoma/nevus) and not a therapy decision (eg, biopsy/reassuring patient). In real life, dermatologists will presumably tend to excise more lesions when they are unsure that the lesion in question is benign, thereby increasing their sensitivity. In a posthoc analysis, dermatologists’ answers were converted from nevus to melanoma when their confidence was low, which showed an increase in excision sensitivity (see Multimedia Appendix 2). In addition, all the images selected for the study were verified by biopsy and are therefore more likely to represent edge cases, which are naturally more difficult to diagnose. This coupled with the nature of the question and the very large test set size could explain the lower diagnostic performance.

A limitation of our current classifier is that it can only distinguish melanomas from pigmented nevi. However, multiple studies have shown that it is possible to create CNN-based algorithms that can distinguish several classes of lesions, thus better reflecting the clinical reality [6,8,23]. Further, the classifier’s performance on an external test set would likely be lower; however, classifier performance was not the primary aspect of this study, but rather if and how dermatologists are influenced by such a system.

### Conclusions

Our results support further research into AI-based classifiers as diagnostic aids in skin cancer classification. We show that clinicians can improve their overall accuracy through improving sensitivity at constant specificity by learning to optimize their interactions with a classifier. While users switched to the correct answer more often than to the incorrect one, minimizing incorrect switches is a challenge that requires further investigation. Our study also has some limitations such as a comparatively artificial setting. In future, clinical trials should be performed to investigate how AI-based classifiers affect skin cancer classification in a real-life setting in which an improved classifier is incorporated in the diagnostic routine.

## Appendix

Multimedia Appendix 1 Detailed methods description.

Multimedia Appendix 2 Additional figures and tables.

## Abbreviations

AI: artificial intelligence
CAD: computer-aided diagnosis
CNN: convolutional neural network
ISIC: International Skin Imaging Collaboration
