# Supplementary material for: Artificial Intelligence and Its Effect on Dermatologists’ Accuracy in Dermoscopic Melanoma Image Classification: Web-Based Survey Study
Source: J Med Internet Res. 2020 Sep 11;22(9):e18091. doi: 10.2196/18091 (PMC7519424; doi:10.2196/18091)
Supplement: Multimedia Appendix 1 [file jmir_v22i9e18091_app1.doc]

**Appendix 1**

*1.1: Classifier training*

A total of 24 classifiers were trained, one for each of the 24 training sets. One randomly picked training set and its corresponding validation set were picked as a representative and used to establish hyper-parameters and the optimal training procedure for all remaining classifiers. After initially training, all 24 classifiers (12 test sets with each consisting of 2 subsets => 24 sets i.e. 24 classifiers) were trained using the previously established training procedure. The classification threshold α, used to map the continuous output probability to a binary value (0=nevus, 1=melanoma), was adjusted to 0.3 (default α=0.5) by establishing overall classifier performance on all validation sets using a range of different classification thresholds. As modern neural networks have a tendency to be poorly calibrated [1], the validation set was also used to ensure that the probability estimates were representative of the true correctness likelihood, and that no further calibration was necessary. Before models were deployed for use in the survey, a final training run of all 24 classifiers was carried out on the recombined training and validation set using the previously established training procedure.

The CNN architecture was a ResNet-50 pre-trained on the ImageNet database. A training run consisted of 20 epochs. Training of the classifier was carried out in five steps, where each step followed Leslie Smith’s 1cycle policy [2]. Additionally, hyper-parameters were adapted in-between each step. Steps 1-4 lasted for four epochs each; with step 5 being two epochs long. Learning rates for the individual steps were chosen and fixed using a learning rate finder which performs a training run of the model where the learning rate starts low and is gradually increased until the loss gets too high. The learning rates were chosen so that they had values were the loss was still decreasing. For the initial two steps, the model was trained on images of 224x224 pixel resolution and for the last three steps the resolution was increased to 448x448 pixels, while reducing batch size by a factor of 4. Original images had a resolution of 600x450 or higher. During training, the network was first trained in a frozen state where only the fully-connected layers were trained at a high learning rate, followed by a step where the complete network was trained using differential learning rates. Earlier layers, which are already pre-trained and fine-tuned to detect general features, such as basic shapes and colour gradients, are trained on low values. The further the layer is from the input, the larger the learning rate becomes until a pre-set maximum is reached. Differential learning rates allow for a stronger modification of the later layers, which represent application-specific features, and therefore need more adjustment than pre-trained earlier layers.

*1.2: Characteristics of the Participants*

The group of participants constituted of eight male and four female board-certified dermatologists. They had a median of 11 years’ experience in dermatologic practice (range 5–30) with seven dermatologists having 3-10 years and five more than 10 years of experience with dermoscopic examinations of skin lesions. The number of carried out skin cancer screenings over the past year (measured in intervals) varied from <50 to >1000 with the median lying between 50-500.

*1.3: Survey Evaluation*

For evaluation, image quality (categorical) was converted into a grade (integer, range 1-5, 1=excellent, 5=other image problems/no image visible). The certainty given by participants for each image (numerical, range 0-10) was interpreted as the dermatologists’ confidence value and treated as percentages by mapping to the range 0-100. Confidence values for the CNN were the output probability of the network for each class, after modification. Of the 1200 rated images, a subset of 67 was removed as they were marked with a score lower or equal to 4 (=inadequate) by participating dermatologists.

**Appendix References**

1. Guo C, Pleiss G, Sun Y, Weinberger KQ, editors. On calibration of modern neural networks. Proceedings of the 34th International Conference on Machine Learning-Volume 70; 2017: JMLR. org.

2. Smith LN. A disciplined approach to neural network hyper-parameters: Part 1--learning rate, batch size, momentum, and weight decay. arXiv preprint arXiv:180309820. 2018.
