# Supplementary material for: Artificial Intelligence and Its Effect on Dermatologists’ Accuracy in Dermoscopic Melanoma Image Classification: Web-Based Survey Study
Source: J Med Internet Res. 2020 Sep 11;22(9):e18091. doi: 10.2196/18091 (PMC7519424; doi:10.2196/18091)
Supplement: Multimedia Appendix 2 [file jmir_v22i9e18091_app2.doc]

**Appendix 2**

**Figure 2.1. Performance of dermatologists broken down for each part (without AI or with AI support) and survey (survey 1 or survey 2).** Top row: results for part I (without AI support) where performance across both surveys is similar. Bottom row: results for part II (with AI support) where performance shows an increase in correct diagnoses for survey 2 compared to survey 1.

**
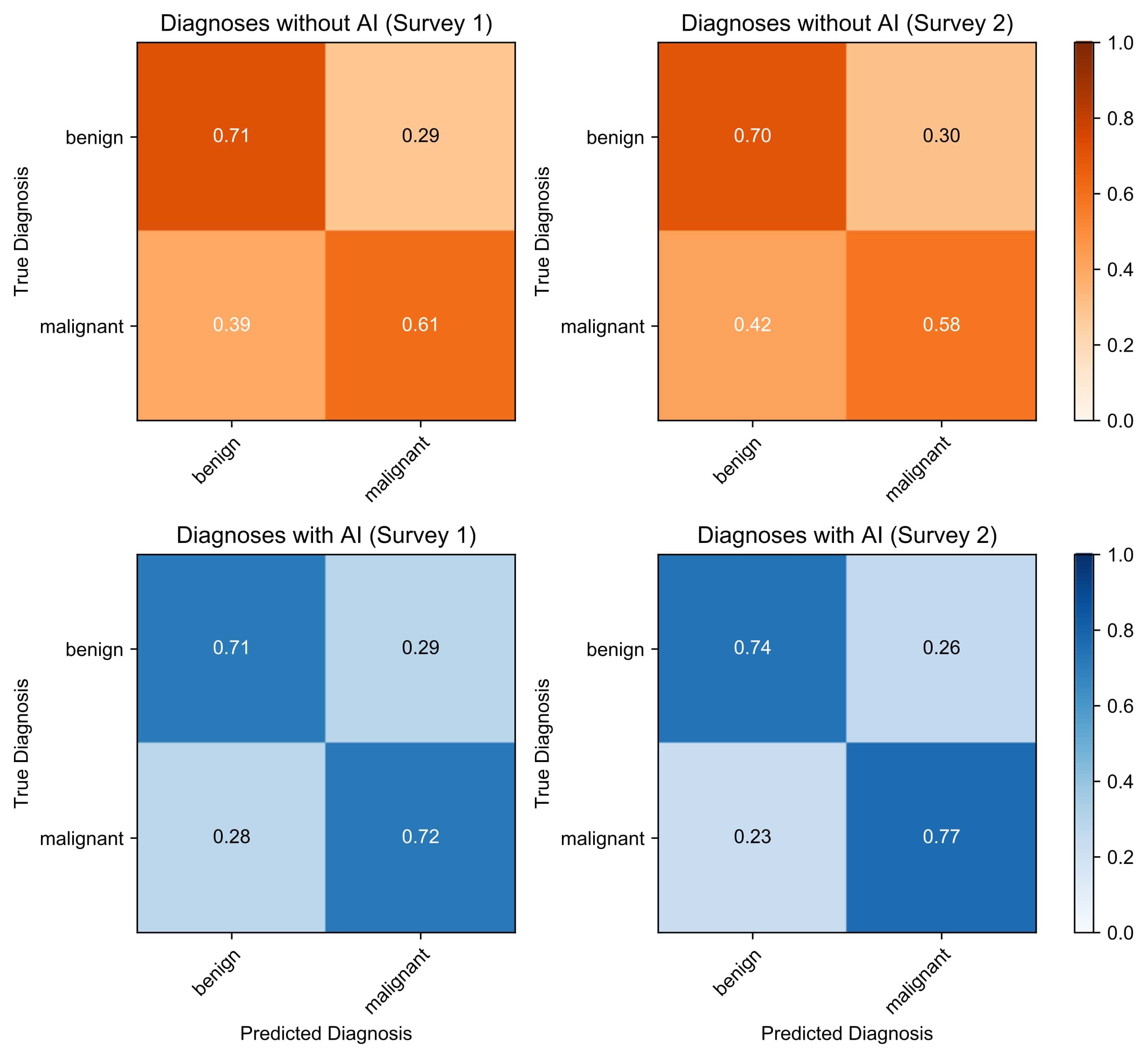
**

**Table 2.2. Post-hoc analysis of dermatologists’ performance without (-AI) and with AI support (+AI) when considering therapy management instead of pure classification.** Sensitivity, specificity, and accuracy were re-calculated after changing all nevus diagnosis coupled with a low confidence value (<50%, 60% or 70%) to melanoma. The highlighted column shows results for the original classification task (unmodified). As the threshold increases, more nevi are classified as melanoma leading to a higher sensitivity at a loss of specificity. These values come with their own limitations as excision based on confidence differs from dermatologist to dermatologist.

|  | **Derm (-AI)** | | | | **Derm (+AI)** | | | |
| --- | --- | --- | --- | --- | --- | --- | --- | --- |
|  | **Original** | **<50%** | **<60%** | **<70%** | **Original** | **<50%** | **<60%** | **<70%** |
| **Sensitivity (95% CI)** | 59.4% | 67.0% | 73.9% | 81.3% | 74.6% | 82.3% | 85.3% | 89.2% |
| **Specificity (95% CI)** | 70.6% | 59.5% | 52.0% | 41.5% | 72.4% | 63.9% | 58.7% | 51.4% |
| **Accuracy (95% CI)** | 65.0% | 63.4 % | 63.2% | 61.8% | 73.6% | 73.3% | 72.3% | 70.8% |
